# P-1684. Characterization of Bacteremia and de-escalation practices among 13 public hospitals in Chile: Results from the 2022-2023 Chilean PPS

**DOI:** 10.1093/ofid/ofae631.1850

**Published:** 2025-01-29

**Authors:** Dona Benadof, Mirta Acuña, Paola Lichtenberger, Tania Herrera, P, Jose Luis Bustos Mejia, Francisco Zamora, Ruth A Rosales, Maria Alejandra Lobos, Loreto Rojas, Rodrigo Orellana, Ricardo J Soto, Catalina C Gutierrez

**Affiliations:** Roberto del rio children hospital, Santiago, Region Metropolitana, Chile; Roberto del rio children hospital, Santiago, Region Metropolitana, Chile; University of Miami, Miami, Florida; Health Ministry of Chile, Santiago, Region Metropolitana, Chile; Hospital Clínica Bíblica, San José, San Jose, Costa Rica; PAHO, Guatemala City, Sacatepequez, Guatemala; Hospital Barros Luco, Santiago de Chile, Region Metropolitana, Chile; Complejo Asistencial Barros Luco Trudeau, Santiago, Region Metropolitana, Chile; Hospital de Castro, Castro, Los Lagos, Chile; Puerto Montt Hospital, Puerto Montt, Los Lagos, Chile; Hospital de Peñaflor, Peñaflor, Region Metropolitana, Chile; Hospital Mauricio Hayermann Torres de Angol, Angol, Araucania, Chile; Hospital San Juan de Dios, Santiago, Region Metropolitana, Chile

## Abstract

**Background:**

Antimicrobial resistance (AMR) is a global concern. Collecting data and implementing interventions to optimize antimicrobial (AM) use has significant potential to lower AMR.There is no information in Latin American Region on how prescribers are using data from positive blood culture results to de-escalate AM. We present a sub-analysis of the 2022-2023 Latin-PPS performed in Chile including the characterization of bacteriemia and de-escalation practices.

Antimicrobial distribution to treat bacteremia

**Figure:**
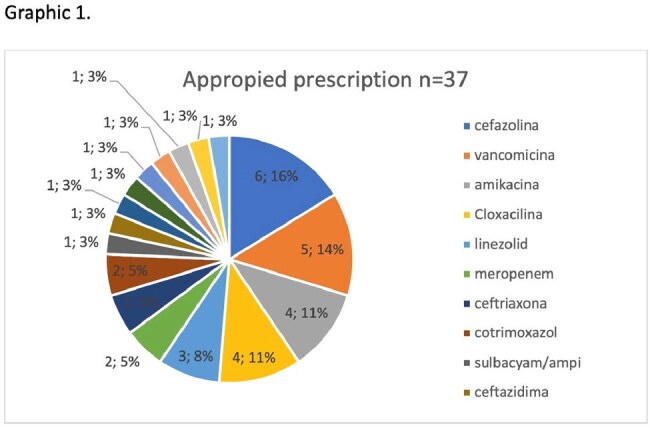

**Methods:**

13 public hospitals joined the Latin-PPS group. This PPS is based on the WHOPPS methodology. Each hospital conducted a survey in which everyday data from AM prescriptions was collected by ward. We include only patients that received AM related to documented bacteremia. The purpose of this study is to describe the etiological agents that caused bacteremia, AM use and compare the blood culture result with appropriate AM prescription.

Most common etiological agents and antimicrobials used.

**Figure:**
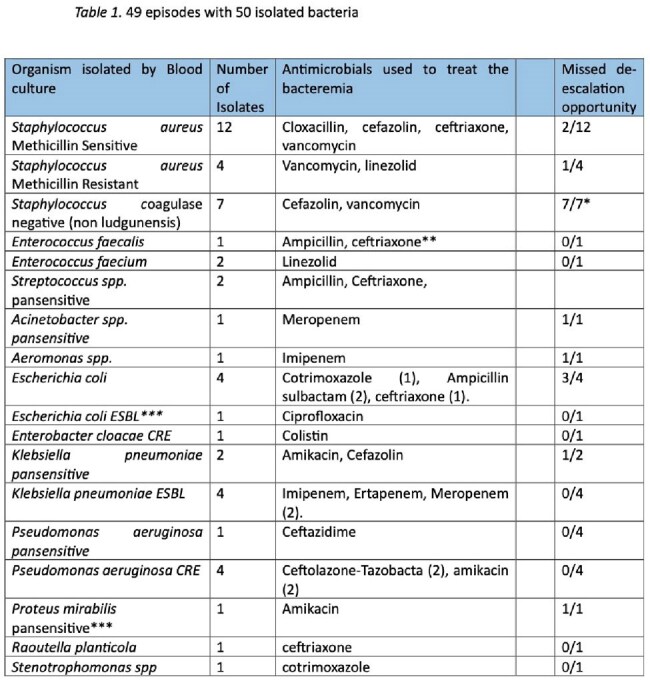

**Results:**

Chilean PPS included 3,342 beds, where 1302 AM treatments were given and 1,928 AM were administered to a national prevalence of 38.84%. Bacteremia is the 6^th^ most common diagnosis related to AM use and corresponds to 3,76% . We found 49 bacteremia episodes, 9 occurred in ICU. The most common etiological agent was *Staphylococcus aureus* (16/49). Table 1 describes: most common etiological agents and AM used. The AM distribution to treat bacteremia is shown in graphic 1.

37/49 (75.5%) bacteremia had an organism that matched an appropriate AM. 10 of those missed an opportunity of de-escalation and 7 possibly represented contaminations in which AM could be stopped.

**Conclusion:**

The AM prescription in hospitalized patients in Chile is lower compared to other LMIC. We found opportunities for AM optimization by de-escalating or stopping AM when blood culture results are available in bacteremia. We plan to recommend evidence-based strategies implementation aiming to de-escalate AM to a narrow more tailored regimen by audit and feedback supported with guidelines. Also to raise awareness about the possibility of contamination when blood cultures isolate coagulase negative staphylococcus as an opportunity to stop antimicrobials. PPS is an effective method to detect antimicrobial prescription habits and prioritize Antimicrobial Stewardship activities with local data.

**Disclosures:**

**Dona Benadof, MD**, Biomerieux: Grant/Research Support **Mirta Acuña, MD**, Pfizer: Grant/Research Support **P. , PharmD**, Eli Lilly: Stocks/Bonds (Private Company)|MSD: Advisor/Consultant|Pfizer: Advisor/Consultant **Ruth A. Rosales,** n/a, BECTON DICKINSON DE CHILE: Advisor/Consultant|BECTON DICKINSON DE CHILE: Speaker|Pfizer SA: Speaker

